# Agent-based analysis of contagion events according to sourcing locations

**DOI:** 10.1038/s41598-021-95336-5

**Published:** 2021-08-06

**Authors:** Mijat Kustudic, Ben Niu, Qianying Liu

**Affiliations:** College of Management, Shenzhen University, Shenzhen, 518060 China

**Keywords:** Computational science, Computer science, Statistics, Computational models

## Abstract

The first human infected with the Covid-19 virus was traced to a seafood market in Wuhan, China. Research shows that there are comparable types of viruses found in different and mutually distant areas. This raises several questions: what if the virus originated in another location? How will future waves of epidemics behave if they originate from different locations with a smaller/larger population than Wuhan? To explore these questions, we implement an agent-based model within fractal cities. Cities radiate gravitational social attraction based on their Zipfian population. The probability and predictability of contagion events are analyzed by examining fractal dimensions and lacunarity. Results show that weak gravitational forces of small locations help dissipate infections across country quicker if the pathogen had originated from that location. Gravitational forces of large cities help contain infections within them if they are the starting locations for the pathogen. Greater connectedness and symmetry allow for a more predictable epidemic outcome since there are no obstructions to spreading. To test our hypothesis, we implement datasets from two countries, Sierra Leone and Liberia, and two diseases, Ebola and Covid-19, and obtain the same results.

## Introduction

As the world struggles with the Covid-19 pandemic researchers are left asking numerous questions. These questions can be directed towards the past, for example, which species are the source of the virus^1^, towards the present regarding what countermeasures should be implemented^2^ or the future, by trying to predict the economic consequences of pandemics^3^.

To prevent similar events in the future we must understand how this virus jumped from bats to humans. The first infected human was traced back to a seafood market in Wuhan, China^4^. Research shows that a similar type of bat lives in Yunnan province and China shares 96% of its genetic sequence with SARS-CoV-2^5^. According to these facts, we must ask a question regarding the starting scenario of the virus: what if the virus originated from another location, with a smaller/larger population than Wuhan?

Research points to a possibility for managing the risk of pandemics following the extreme value theory (EVT) manifested through power laws^6^. It considers that extremes and not averages are fundamental sources of risk. An exploration of these phenomena has prompted the use of fractal geometry^7^ and fractal reaction principles^8^. In simple terms, fractals are pattern-like shapes that can be seen in snowflakes, lightning, clouds, and numerous plants such as broccoli or ferns. There is proof that this pandemic follows some fractal structure rules and shows a similar pattern in different regions of the world^9,10^.

The first question in this paper examines disease dynamics and cross-country disease spreading when the disease originates from different sized source populations. The second question analyses event probability, predictability, and emergent behaviors of agents as they navigate across cities.

To answer these questions, we use two main methodological innovations and approaches. The first one comes from implementing an agent-based compartmental, and reservoir arrangement of fractal cities. All cities radiate gravitational social attraction based on their Zipfian distributed population. The second methodological innovation is based on implementing fractal dimensions and lacunarity for analyzing event probability and its consequences.

To test our hypothesis, we implement real datasets from two countries, Sierra Leone and Liberia, and two diseases, Ebola and Covid-19. Ebola started in the rural areas with a small populace^11^ while Covid-19 started in China^4^ and was imported to the capital cities of respected countries. This difference in starting location makes for a perfect ground to test our hypothesis.

The remainder of the paper is structured as follows. Section 2 focuses on reviewing literature according to different disease modeling approaches. Section 3 describes the implemented framework based on multi-dimensional spatial distribution coupled with a compartmental aspect. Section 4 describes experimental results while Sect. 5 shows how real diseases spread according to obtained datasets. Section 6 discusses and compares predicted with real disease dynamics. Section 7 discusses future research directions and concludes the paper.

## Literature review

### Disease modeling approaches

Disease modeling is a useful tool that can give insight into disease dynamics so that an effective response can be developed. The basic notions were defined by^12^ through their compartmental model that uses linear equations. New and more flexible approaches often implement some variant of artificial intelligence one of them being agent-based modeling (ABM). The modeled disease can be unspecified^13^ or use exact pathogen characteristics, such as the case of Ebola^3^ or Covid-19^14^. Observations can be made according to the governmental action and individual reaction^15^, self-initiated preventive actions^16^, or according to disease-carrying vectors^17^. AB models have been used to define country-level epidemiological control and prevention measures^18^, recently a strategy developed in this manner^2^ has been implemented in the United Kingdom. An important topic regarding Covid-19 is the research of the economic effects of crises caused by countermeasures such as lockdowns^19^. Research can go into such details to explore how better handwashing can significantly affect epidemiological outcomes^16,20^.

### Role of geographical factors on disease spreading

Epidemics can be viewed as diffusion waves and should be modeled similarly to other spread and change processes^21^. Therefore, the key factors for disease spreading can be narrowed down to the spatial (geographical) factors, human behavior, and time dependence factors^22^. When observing distances between cities or countries, a strong explanatory potential for their populations’ behavior can be found in the models that are based on Newton’s gravity law, where the sizes of these elements play the role of mass^23^. Smaller cities are characterized by a smaller population, however, on average, all cities have similar sizes of social groups^16^. In general, large cities have a greater probability to become large spreaders compared to smaller locations because of their international connectedness and tight commuting relationships^24^.

In standard economic practice, this form has been used for analyzing migration^25^, urban population density^26^, transportation^27^, and so on. The gravitational approach is commonly implemented along with epidemiological models for disease prediction or analysis where the observation focus can be on different diseases, such as measles^28^ and influenza^29^ or to observe disease waves, and their spatial hierarchies of concentration^30,31,32^. Analysis can be focused on urban disease spreading^33^ and discovering possible solutions for its suppression^18^. Due to Covid-19’s prevalence, containment needs to be done by implementing non-pharmaceutical measures such as lockdowns to prevent cross-country spreading^2^.

### Fractal patterns of diseases

One approach for managing limitations of modeling comes by observing event probabilities and their “contagion event sets”^9^ where the idea of fractality greatly helps. Due to the relative mathematically based similarities with epidemiological modeling, pandemics caused by Covid-19 have conceived some fractal observations. Repeating patterns, power-law behaviors and scaling properties across different regions of the world have been noted^10^. Scalability is also explored through different sized social networks and clusters pointing out the fractal dimensions^9^. We note a definite lack of literature in this area requiring further exploration due to the present and future dangers of the Covid-19 pandemic as well as other possible ones.

An important class of models that introduces realism through heterogeneity is based on metapopulations^33^ also referred to as fractality^10^. These models divide the network of agents into subpopulations of different sizes. This feature is important because it allows for the implementation of cities of different sizes which is an important aspect because it sets the limit on how large a fraction of the population a single individual might meet within a given period^34^.

**Figure 1:**
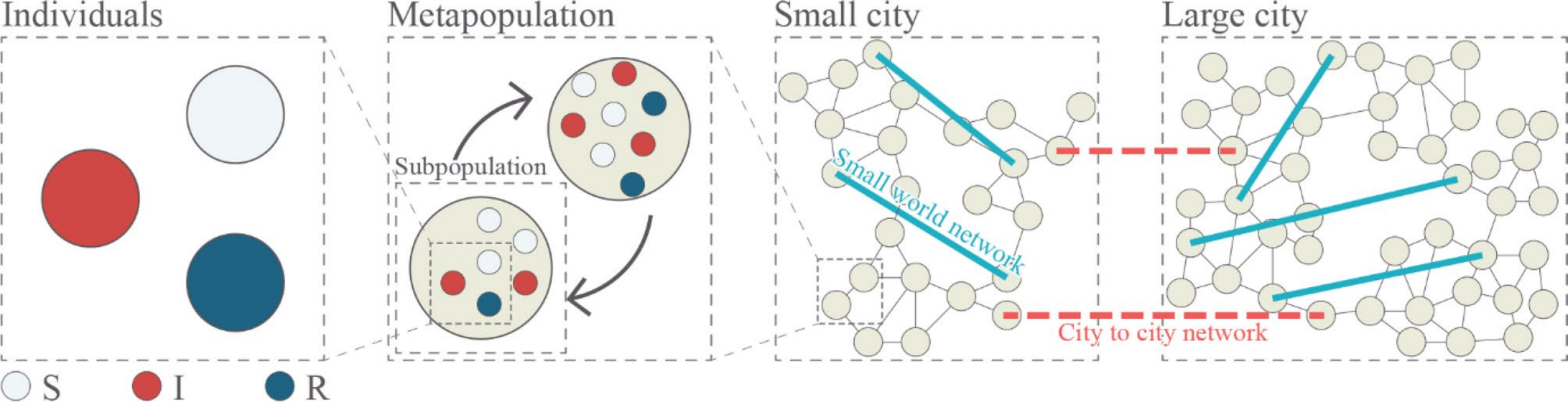
Shows different sized cities acting as reservoirs, they are distributed across a country. A bottom-up approach is obtained by implementing individuals, metapopulation, and subpopulations within cities. Small-world networks are implemented for obtaining circulatory dynamics.

There are two main reasons why the epidemic spreading is likely to follow a fractal pattern, both in line with the main characteristics of fractals, recursiveness, and self-similarity. Recursiveness can be traced to the underlying framework of epidemics, namely the population, which tends to follow a power-law function in its self-organization^35^. These aspects of human activity in urban centers have been analyzed in the science of cities that show how people live and interact in urban environments. The reason for recursiveness lies in the scalability of spreading where the transmission dynamics of the individual level (Fig. 1) is the same as the one in the subpopulation, metapopulation, or city to city level^10^. Since all of these levels have the same transmission dynamics it leaves the scale of observation invariant, which is in line with the inherent behavior of cities^35^.

Self-similarity patterns of the epidemic appear all over the world and have been identified in Romania, Italy, Spain, Germany^36^, as well as in China, the USA, Brazil, and Europe^10^. This self-similarity of epidemics can be viewed as a useful feature because it allows researchers to assess the current condition and predict the next modifications in the epidemiological curves^36^.

## Framework description

The used framework consists of two parts, the spatial element, and the compartmental element. The spatial part is used to observe the movement and dynamics of the population and their cross-city behavior during epidemics. The compartmental element enables pathogen transmission and its observation.

### Spatial dynamics of fractal cities

People can live in a city for a long time, meeting some inhabitants often and others, not at all^10^. This isolation feature of social clusters prevents the exponential spreading of diseases since greater numbers of infected do not directly correlate with a greater probability of getting infected. Therefore, the probability of infection refers more to social groups and not individuals because through an individual the entire group of closest contacts is in contact with another group.

Although the fractal scale range can be infinite, we define the lowest scale value as an individual agent. This agent is an integer and is in one of the compartmental states ${ }S,I{\text{ or }}R$. A higher scale of observation comes from incorporating the surrounding metapopulation and subpopulation of multiple cities within a country, as defined by^10,33^. Agents are distributed across social groups of different sizes with the average one having 10 close individuals^16^. Figure 1 Shows the highly clustered network with a short average path length that is also highly intertwined hence it also can be defined as a network with a small-world structure (SWN)^37^.

Cities are geographically and epidemiologically separate with the distance between them acting as a barrier inhibiting disease transmission. Since this observation coincides with the definition of epidemiological reservoirs^38^ we use the same terminology. There are several population types based on the location of infected individuals. If there is an infected individual inside a city and the disease spreads within it, the population (city) is considered as host. Population from which the infection starts, meaning that it transmits the infection directly to another population, we define as a source population. The target population is the population of interest to the observer.

### Compartmental organization

The compartmental organization is based on the SIR model^12^ that consists of susceptible ($S$), infected ($I$), and recovered ($R$) individuals, where the $S$ can become $I$ while the $R$ does not return to the previous stages $\left( {S_{t} \to I_{t} \to R_{t} } \right)$. Models may incorporate more compartments (quarantine, treated, or vaccinated individuals) as well as recurring movement across compartments. The total population of each model is considered to be constant, as noted:

1 $$ N = S_{t} + I_{t} + R_{t} $$

where the total population is ${ }N$, note that we incorporate time $t$ since the compartments may differ while still having susceptible ($S_{t}$), infected ($I_{t}$) and recovered ($R_{t}$) individuals. Formulas presented next define change between compartments:

2 $$ S_{t} = - \beta S_{t} I_{t} $$

3 $$ I_{t} = \beta S_{t} I_{t} - \gamma I_{t} $$

4 $$ R_{t} = \gamma I_{t} $$

So that $\beta { }$ shows the rate of infection, while $\gamma$ is the removal rate of infected individuals. City observations are treated percentage-wise since commuting from $(N_{c}^{o} )$ and to $\left( {N_{c}^{d} } \right)$ a city due to mutual gravitational forces $\left( {F_{c} } \right)$ changes the population of the city $N_{c}$ but the country $N$ stays constant. The city-specific susceptible population can be calculated as:

5 $$ S_{t} = \mathop \sum \limits_{c = 1}^{cities} S_{t}^{c} $$

This formulation defines $S_{t}$ as a function of all susceptible individuals spatially distributed across cities at a certain moment, other compartments use the same principle. To define and distribute the population size we use Zipf’s law^39^. To calculate the population of each city we use the following equation:

6 $$ f\left( {k;s,N} \right) = \frac{1}{{k^{s} H_{N,s} }} $$

So that $N$ is the total population size of a country, $k$ is the rank of the city and $s$ exponent represents the value that characterizes the distribution which is 1.07 according to^39^. The calculation begins with the largest city and percolates to smaller cities so that the $n$th the city population is the $\frac{1}{{{ }n^{s} }}$ of the largest city. Varying population sizes can depict different phenomena by implementing a multilayered observation instead of a node-based one.

Different population sizes are important for predicting population movement, as described by the law of demographic gravitation^40^. The law is based on Newtonian principles of distance and mass and explains how cities have attraction forces that draw individuals to migrate or visit them, as shown by the following formula:

7 $$ F_{c} = \frac{{N_{c}^{o} {*}N_{c}^{d} }}{{d^{2} }} $$

where $F_{c}$ presents the force of attraction, $N_{c}^{o}$ is the size of the city population of origin, $N_{c}^{d}$ is the destination city population and $d$ is the distance between the two cities. Cities interact via migrating agents and daily migrations do not influence their overall gravitational attraction.

### Fractal dimensions

Natural and biological features are often fragmented implying the existence of a fractal dimension. Since for each naturally occurring fractal, there is a finite scaling range (zoom), the structure can become smooth (Euclidean) or rough and random (non-self-similar). In Euclidean n-space, a bounded set $S$ can be considered statistically self-similar if $S$ is the union of $N_{r}$ non-intersecting subsets for a scaling factor $r$, each of which is of the form $r$($S_{n}$) where the $N_{r}$ and $S_{n}$ sets are congruent in distribution to $S$. We can use the fractal dimension to measure $S$ the same way as we use a measurement tool in the Euclidean (discrete) space^7^, it is calculated:

8 $$ FD = \frac{{\log N_{r} }}{{\log \frac{1}{r}}} $$

So that $N_{r}$ is the number of self-similar (invariant) shapes and $r$ is the corresponding scaling factor. On the Hausdorff Dimension scale (HD), a smooth line has a dimension of 1 which is a low value while the high values are present in the Mandelbrot or the Julia set with the complexity of 2. Thus, often the fractal dimension is 1 < HD < 2.

### Fractal dimension calculation

To understand the complexity of a shape we use fractal dimensions as a measure, for calculating it we will use the “Minkowski-Bouligand dimension,” or the “box-counting method”, its pseudocode is presented in Table 1. Calculations can be made with different sized boxes for more or less accurate representation potentially giving different results^41^. The number of boxes, for proportion, used in this paper is 160 obtaining a fractal dimension of 1.25 as in^41^. The calibration image is listed in references^42^.

**Table 1:**
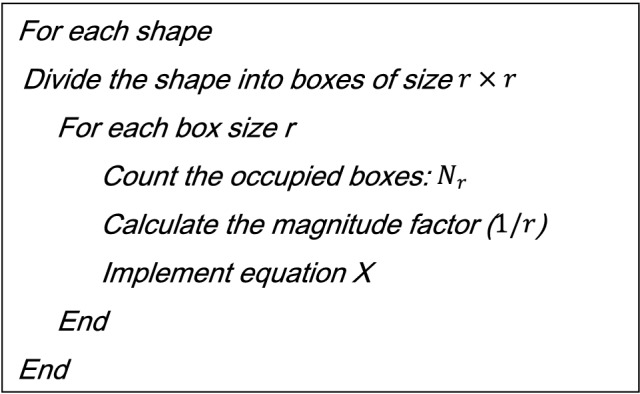
Pseudocode for the box-counting approach for calculating fractal dimensions.

With lower HD numbers we examine the speed and impact of the infection, a single wave it will be shown by lower numbers. If it is rebounding and multivalve it will show more complexity and thus greater numbers. Another dimension is observed through the total mean (cross country) HD where higher numbers show greater dynamics and more diversified results. To further differentiate results, we will calculate lacunarity which is a measure of the gap structure in patterns and coincides with abrupt declines in dispersal success on fractal landscapes^43^. It can be also be viewed as a measure of “gappiness” and heterogeneity, higher numbers showing greater emptiness. Lower numbers imply a slow and steady infection while higher ones a dynamic and/or rebounding one.

9 $$ {\text{L}} = { }\frac{{1/MN\mathop \sum \nolimits_{m = 0}^{M - 1} \mathop \sum \nolimits_{n = 0}^{N - 1} I\left( {m,n} \right)^{2} }}{{\left( {1/MN\mathop \sum \nolimits_{k = 0}^{M - 1} \mathop \sum \nolimits_{l = 0}^{N - 1} I\left( {k,l} \right)} \right)^{2} }} - 1 $$

It is defined in terms of the ratio of the variance over the mean value of the function where $M$ and $N$ are the sizes of the HD for the processed graph (image)^44^. We will use it for analyzing the numbers of infected.

### Experimental parameters

Table 2. shows experimental parameters that are applied for all scenarios. Implemented disease characteristics are based on^15^ with the infecting probability being 5% per contact and disease duration 10 days. The population of the largest city is trickled down by using Eq. 7 to define the populations of other cities.

**Table 2:** Parameters for the simulations.

$c$	Number of cities	**10**
	Largest city population	10.000
$N$	Total Population	42,811
$k$	Zipf exponent	1.07
	Default nr. of connections	20

**Figure 2:**
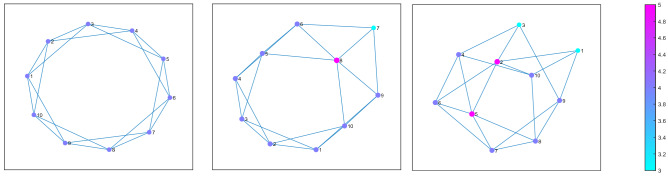
Spatial distributions of the three scenarios. Each scenario figure shows the sizes of the cities, by circles, and by the color bar on the right. Lines present the connections between cities that the agents may use for commuting.

The number of simulation runs for each scenario (3) and each source (2), giving 6 in total, is 50 with each run having 350 iterations. For the spatial component, we use a 10-city setting. Since spatial organization and interconnectedness play an important role, we use three different scenarios as in Fig. 2 with the average network length of 1.5, 1.48, and 1.4 respectfully.

The first scenario is based on an SWN with high interconnectedness of cities, as in^37^. All cities are equally distant from one another, no city is better connected or presents a networking hub, the only difference is the population distribution. The second scenario has a semi-circular SWN orientation. Some cities are more isolated from the largest one, making them more difficult to reach. The third scenario has better connectedness across all city sizes and is more random in its orientation than the first two. Each city is easier to reach than in other cases.

## Experimental results

To discover emergent behavior, we compare intra-scenario results. We check cross scenario results to see how disease spreading is affected by the interconnectedness of cities. We observe the maximum number of infected at a single moment which shows the burden on the health system. The standard deviation of infected population presents the dynamics of infecting. With greater incidence comes greater deviation. The total number of infected is a key indicator of the epidemic spreading, although alone it doesn’t show the timeframe and dynamics.

**Figure 3:**
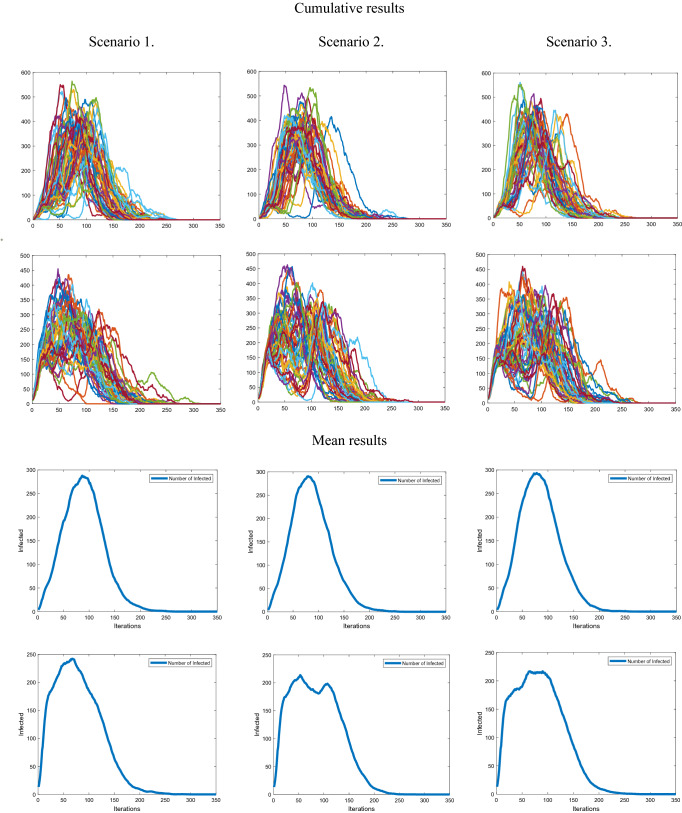
Shows Graphical results of simulations. Top to bottom for both cumulative and mean results are according to the sourcing (starting) positions of the pathogen, smallest and largest city, respectively.

Figure 3 is divided into two parts, the top part displays cumulative simulation results (each graph has 50) while the bottom contains the mean results of all simulations respectively. From left to right different scenarios are shown (1–3) while top to bottom is the sourcing positions of the pathogen, that being the smallest or largest city, respectively.

### First scenario

We see that the situation is less favorable when sourcing from the smallest city, compared to the largest one. Furthermore, the mean HD shows that the dynamics is more predictable and “gravitates” towards the same outcome. Higher lacunarity numbers show the changing incidence rates indicating complexity and dynamics. There is a noticeable jump when infected advance from a small full reservoir to a new large one, similar to the “honeymoon effect” phenomenon^45^ when effective control is used against an endemic infection resulting in an initial drop in prevalence to well below the endemic level. Afterward, it is followed by outbreaks that periodically increase prevalence above the endemic level as a consequence of a build-up of susceptible individuals^46^.

Greater infection intensity depletes the susceptible population eradicating the disease quicker. “In smaller populations, the number or density of infected hosts frequently falls to low levels, random extinction (fadeout) becomes inevitable, and the pathogen cannot persist”^38^. Smaller sites are monitored and tested less often. The potential benefit of a disease spreading from a small location is that it is easier to control and put on lockdown blocking further propagation.

### Second scenario

We see that the smallest city as a source causes higher maximum infected, standard deviation, and the total number of infected. The reason for this comes from the gravitational pull $F_{c}$ of a large city since $F_{c}^{Large} > F_{C}^{Small} { }$ when squared distances are adequately the same, according to (Eq. 7). It will attract potential commuters from smaller cities around its vicinity and will keep the natives within it. Migrants from (other) smaller cities will return to their place of origin and locally spread the disease. The small reservoir is filled quicker so the infection spreads faster across smaller populations. This is combined with the higher probability of people moving from smaller to other cities, due to the small gravity, additionally increasing chances of spreading.

### Third scenario

This scenario shows a lower number of max infected along with a lower standard deviation. Higher interconnectedness allows for disease to spread evenly across a country, so its moment of infection peaking is not concentrated. Although dispersed, it gives the same total number as in other scenarios. When the disease sources from a large city its population presents a large pool for potentially infected individuals. As its gravitational pull attracts individuals from other smaller locations they too participate in the social interactions of the large city and commute back to their place of origin which will help propagate the disease top-down. Due to the Zipfian distribution^39^ (Eq. 6.) more small cities are making it more likely for people to commute from small to a large city.

### Cross country comparison

Differences between scenarios are observed via the maximum number of infected at a single moment and the standard deviation values while lacunarity shows the influence of gravity. Unpredictable outcomes come from the second scenario because the lower network connectedness keeps the infection within a city. Higher connectedness helps spread the disease quicker^37^ as in the third scenario. This combined with less isolation of other cities gives more predictable outcomes when the infection is sourced from the largest city because there are fewer boundaries for disease spreading so the outcomes are difficult to change and therefore influence.

**Figure 4:**
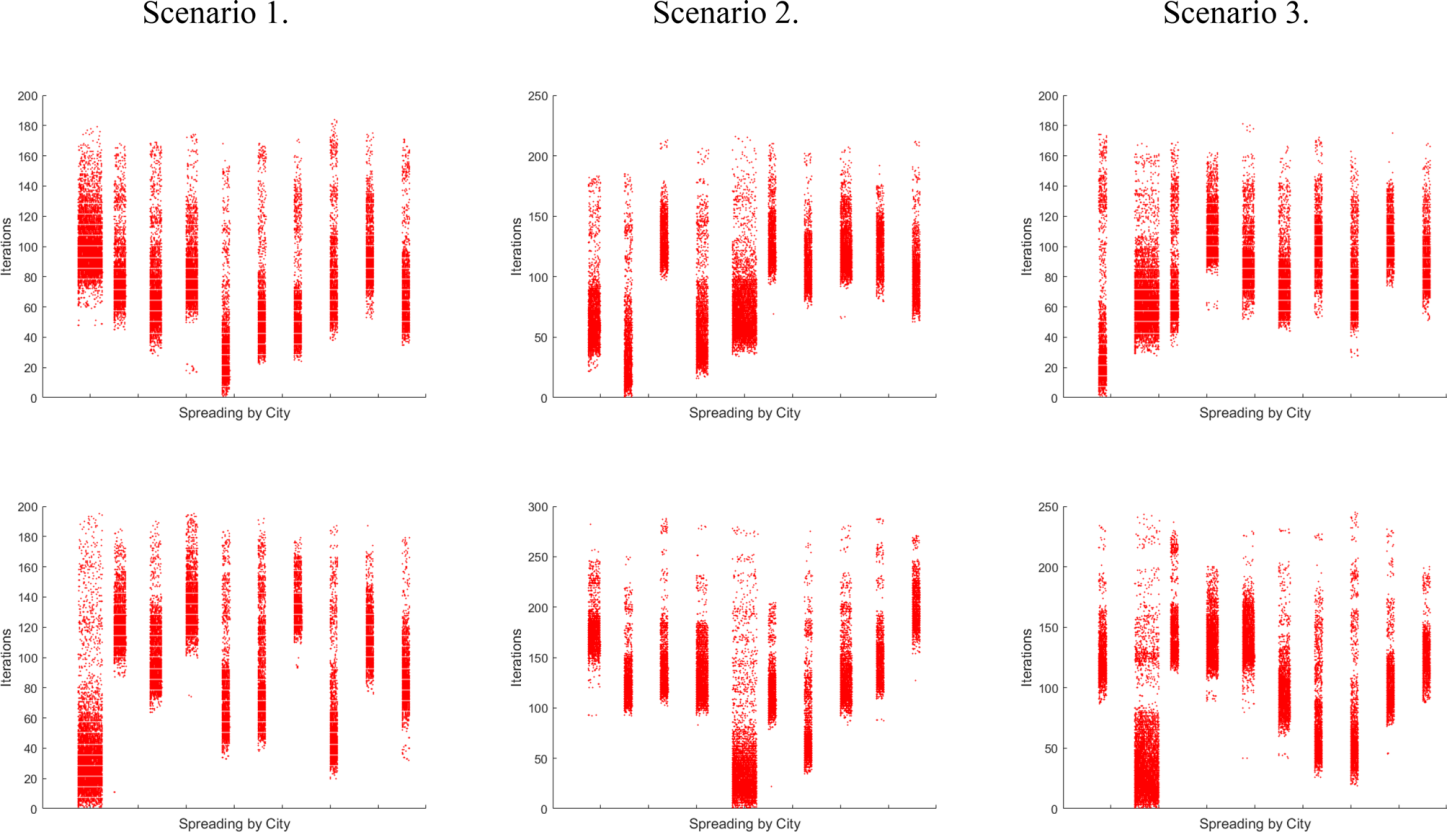
Shows the dynamics of simulations. Top to bottom results is according to the sourcing (starting) positions of the pathogen, smallest and largest city, respectively.

Figure 4 shows simulation dynamics and cross-country disease spreading. A country is divided into 10 cities, width presenting the population, agents are tracked by their daily interactions. The results depict a single simulation.

In Table 3 When observing differences between HD in the 1st scenario we see lower values when the smallest city is the place of origin, pointing to the self-similarity of results. It means that this scenario is the most often repeated, and therefore probable. Greater lacunarity points to spikes of infected at a certain moment, they can be seen via the maximum infected at a single moment. Both are present in every scenario with the small city as the origin. This points to greater incidence due to the infection advancing from a small reservoir to a new large one^45^, also observable through the standard deviation values. By performing the same simulations on more cities with different spatial orientations and population numbers we obtained the same results, note that initial scenario conditions are important.

**Table 3:** Numerical results of simulations.

	Scenario 1		Scenario 2		Scenario 3	
Source City	Small	Large	Small	Large	Small	Large
Maximum Infected	287.78	242.22	300.84	293.52	213.68	216.92
Standard deviation of Infected	100.0444	90.3565	105.3512	102.5832	84.2937	86.1186
Total Infected	27,471	27,465	27,552	27,609	27,656	27,585
Hausdorff Dimension	1.2374	1.2727	1.2365	1.24	1.279	1.2782
Lacunarity	2.1112	1.775	2.1042	2.0317	1.6787	1.6569

## Comparison with real datasets

To test our hypothesis, we implement real datasets from two countries, Sierra Leone and Liberia, and two diseases, Ebola and Covid-19. Ebola started in the rural areas with a small populace while Covid-19 started in China was imported to their capital cities. This difference in starting location makes for a perfect ground for test our hypothesis. Our AB model observes cities while datasets observe districts. Characteristics of the two diseases will be examined in the discussion part.

**Figure 5:**
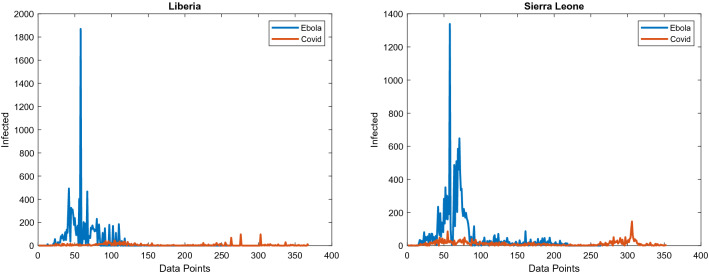
Shows the greater incidence of Ebola compared to Covid-19. Note that the epidemic of Ebola datapoints cutoff while the Covid-19 continue, the reason being is that the outbreak of Ebola lasted from 25th of March 2014. until the 30th of November 2015. Covid-19 epidemic datapoints start from the 15th of March 2020 until the 16th of March 2021, with an ongoing pandemic.

The dataset used for Covid-19 in Liberia is based on the reports published by the National Public Health Institute of Liberia. For Sierra Leone, the data is according to the reports by The Ministry of Information and Communication. The data for Ebola is based on the Ebola World Health Organization (WHO) situation reports for respected countries. Figure 5 shows new cases of Covid-19 and Ebola in Liberia and Sierra Leone cumulatively.

**Figure 6:**
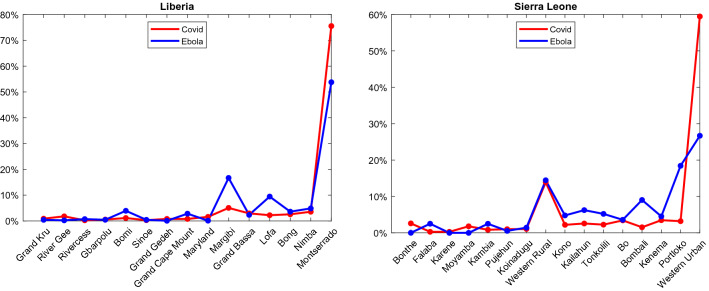
Observes the distribution of infected across country districts, the numbers are presented as a percentage of the total number of infected for easier comparison. The figure points to Ebola being more dispersed across districts, which is observable point by point and percentage-wise.

Figure 6 observes the distribution of infected across country districts. Districts are sorted according to their population, with the least populated on the left, country capital cities are in the most populated districts. The starting point for Ebola was in a rural of Guinea in Guéckédou district^11^, neighboring districts Lofa (Liberia) and Kailahun (Sierra Leone).

Covid-19 is much more present in the capital districts of Montserrado and Western Urban with high numbers appearing in their closest districts, Margibi and Western Rural respectfully. The Lofa district (Liberia) is fitting for disease transmission due to its proximity to the location of disease origin. Secondly, it has a well-developed road network further increasing dissemination^27^.

**Figure 7:**
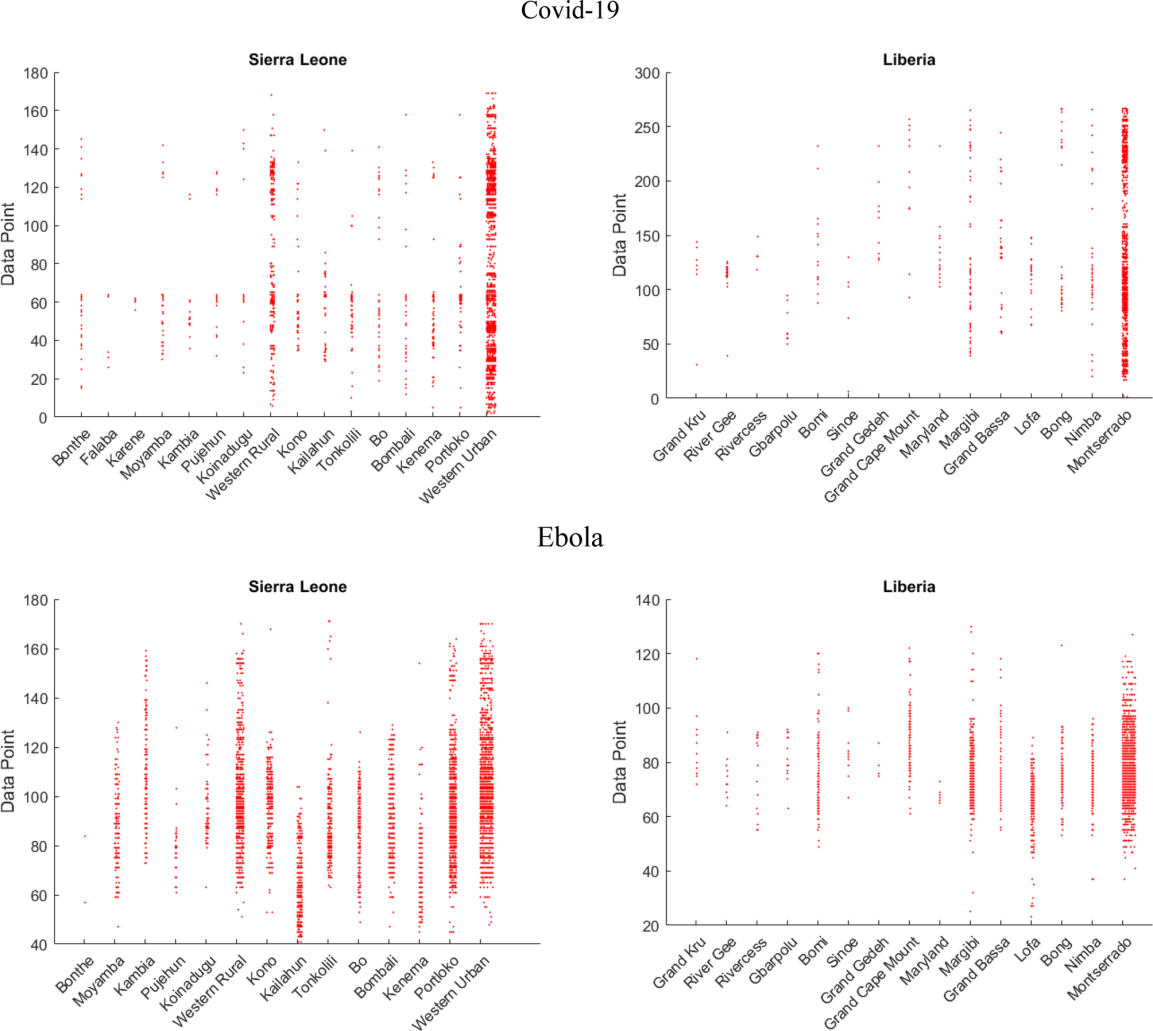
Shows the frequency of newly reported cases in Liberia and Sierra Leone across their districts.

Figure 7 shows that even though there are sparingly new cases in other districts the majority is located in the initial, largest, ones keeping to their gravitational attraction. Regarding Ebola, the reservoir nature of districts can be seen in Kailahun and Lofa. We see that the reservoir contains newly infected individuals for some time before the disease spreads to other districts.

Table 4. shows numerical standpoints of diseases. Our focus is on fractal characteristics for discerning dynamics enabling the prediction of future epidemiological curves^36^. Low HD values of Ebola in both countries point to self-similarity and combined with high lacunarity point to high disease dynamics at a certain point, as seen in Fig. 3. For Ebola in both countries, the initial spike peaked with the maximum number of infected and was not repeated. Covid-19 has lower lacunarity values showing no significant spikes of infected. Higher HD values show an existing turmoil and dynamics of susceptibility to infection.

**Table 4:** Numerical results of diseases based on real datasets.

Disease	Ebola		Covid-19	
Country	Liberia	Sierra Leone	Liberia	Sierra Leone
Maximum Infected	1870	1339	97	146
Standard deviation of Infected	137.24	127.91	10.725	14.794
Total Infected	10,678	14,124	2042	3947
Hausdorff Dimension	1.1403	1.1535	1.296	1.1309
Lacunarity	14.02	14.953	6.1716	3.6046

## Discussion

Demographic gravitation has been used for analyzing disease spreading the focus was not on their potential for containment. Although diseases used for testing our hypothesis are not the same, they present the most documented and concentrated disease (epidemic) observation dynamics in recent decades.

Covid-19 and Ebola are Zoonoses, infectious diseases that originate from wildlife, which represents 60% of known emerging infectious diseases with their numbers growing fast^3^. Both of them most likely originated from bats^5,11^. Differences between the observed diseases should have led to a different turn of events. Theoretically, these differences should enable Covid-19 to be more prevalent than Ebola because it is considerably more transmissible^3^. Being exposed to speaking or coughing is more common than to blood or secretions, which are necessary for the transmission of Ebola. Individuals might be asymptomatic and infectious while Ebola patients are not contagious until they develop symptoms^47^. Greater lethality of Ebola^3^ should have slowed down its progress. Ebola aftermath shows that it has hit more districts and has spread quicker. Even though Covid-19 has started from the largest reservoir it still has not reached its prevalence as Ebola did.

Disease sourcing from rural areas points to behaviors described by our model, those being higher values of lacunarity and maximum infected indicating spikes of infected at a certain period. Ebola also has low HD values in both countries pointing to self-similarity, which is also evident in our simulations. Covid-19 is sourced from capital cities and has lower lacunarity values and no significant spikes of infection. Higher HD values show an existing turmoil and dynamics of susceptibility to infection. The majority of infected are located in the initial cities which keeps to our notion of their gravitational attraction.

Research shows that connectivity matters more than density in the spread of the Covid-19 pandemic^24,48^. Large cities have a greater probability to become large spreaders compared to smaller locations, namely because of their international connectedness and tight commuting relationships^24^. On the other hand, porous land borders, as in the observed countries^49^, enable more population movement compared to airports.

In the case of this paper, both diseases have been introduced from abroad but to different sized locations. This puts high emphasis on connectivity and commuting, but the discussion regarding commuting must be linked with its direction. Demographic gravitation explains that large numbers of people act as an attractive force towards other people to migrate in that direction which is further strengthened by economic factors^48^. This is in line with our gravitational observation where small locations that have less attractional force are not able to stop people migrating towards larger locations. Large locations keep individuals within them due to the same force. When daily commute is introduced individuals from smaller locations more often go to large ones than otherwise. All locations have similar sizes of social groups^16^ because they depend not on the size of the location but individuals. When an infected is introduced to a small reservoir, the reservoir is filled quicker because of the greater infection probability per capita, as can be seen in several locations^50,51^. Now, this small reservoir has greater per capita infection probability, and individuals that are drawn by the greater attraction force of larger locations making them a more dangerous place for the infection to start from.

There are several limitations to our study. Disease spreading is influenced by other important factors that we did not describe. Two diseases under scrutiny are not the same and behave differently in different locations. Although the current situation points to our findings being correct, the Covid-19 pandemic is ongoing and the numbers will continue to change. Circumstances in Liberia and Sierra Leone do confirm our findings but they are relatively small-sized and disease dynamics might not fold the same way in other countries. Finally, our findings are based on numerous simulations which are averaged. Real events happen once and can come out of the most unpredictable sources. Nonetheless, our findings point in the same direction.

## Conclusion

In this paper, we combine a spatial agent-based model with a compartmental (SIR) model to observe multi-city and cross-country epidemics. We observe that gravity maintains the infection inside the city when the sourcing position of the pathogen is the largest city. Sourcing from the smallest city quickly dissipates the infection across a country. To test our hypothesis, we implement datasets from two countries and two diseases. Disease sourcing from rural areas points to the same behaviors as described by our model.

Our future research will be based on implementing different scenarios regarding infection transmission rates and source population locations expanding them to cities of various sizes and levels of connectedness. We will continue to monitor the Covid-19 pandemic and will compare outcomes with our predictions.
